# Impact of *TIEG1* Deletion on the Passive Mechanical Properties of Fast and Slow Twitch Skeletal Muscles in Female Mice

**DOI:** 10.1371/journal.pone.0164566

**Published:** 2016-10-13

**Authors:** Malek Kammoun, Philippe Pouletaut, Francis Canon, Malayannan Subramaniam, John R. Hawse, Muriel Vayssade, Sabine F. Bensamoun

**Affiliations:** 1 Biomechanics and Bioengineering Laboratory, UMR CNRS 7338, Sorbonne University, Université de Technologie de Compiègne, Compiègne, France; 2 Department of Biochemistry and Molecular Biology, Mayo Clinic, 200 First Street SW, Rochester, Minnesota, 55905, United States of America; University of California, Davis, UNITED STATES

## Abstract

As transforming growth factor (TGF)-β inducible early gene-1 is highly expressed in skeletal muscle, the effect of *TIEG1* gene deletion on the passive mechanical properties of slow and fast twitch muscle fibers was analyzed. Twenty five muscle fibers were harvested from soleus (Sol) and extensor digitorum longus (EDL) muscles from *TIEG1*^-^/^-^ (N = 5) and control (N = 5) mice. Mechanical tests were performed on fibers and the dynamic and static stresses were measured. A viscoelastic Hill model of 3rd order was used to fit the experimental relaxation test data. In parallel, immunohistochemical analyses were performed on three serial transverse sections to detect the myosin isoforms within the slow and fast muscles. The percentage and the mean cross sectional area of each fiber type were calculated. These tests revealed a significant increase in the mechanical stress properties for the *TIEG1*^-^/^-^ Sol fibers while a significant decrease appeared for the *TIEG1*^-^/^-^ EDL fibers. Hill model tracked the shape of the experimental relaxation curve for both genotypes and both fiber types. Immunohistochemical results showed hypertrophy of all fiber types for *TIEG1*^-^/^-^ muscles with an increase in the percentage of glycolytic fibers (IIX, and IIB) and a decrease of oxidative fibers (I, and IIA). This study has provided new insights into the role of *TIEG1*, known as KLF10, in the functional (Sol_typeI_: more resistant, EDL_typeIIB_: less resistant) and morphological (glycolytic hypertrophy) properties of fast and slow twitch skeletal muscles. Further investigation at the cellular level will better reveal the role of the *TIEG1* gene in skeletal muscle tissue.

## Introduction

Transforming growth factor (TGF)-β inducible early gene-1 (*TIEG1*) was originally identified as a primary response gene following TGF-β treatment of human osteoblasts [1]. The *TIEG1* mRNA is known to be induced within 30 minutes of growth factor treatment, with a maximum induction occurring by 90 minutes. At the protein level, *TIEG1* is induced maximally between 2–3 hours following growth factor treatment. The expressed *TIEG1* protein is very labile as it is targeted rapidly for proteosomal degradation by the E3 ubiquitin ligase, SIAH [2]. *TIEG1* is a member of the Krüppel-like transcription factor family (*KLF10*) and contains three C2-H2 zinc fingers at its C-terminal end [1]. *TIEG1* is expressed in a wide variety of tissues with maximal RNA expression being observed in skeletal muscle [1, 3–7]. *TIEG1* is a key mediator of TGF-β signaling [8–10] and overexpression of *TIEG1* in multiple cell types mimics TGF-β actions [5, 6, 11].

It has been shown that deletion of *TIEG1* affects the development of bone [12] and tendon tissues [13] and that *TIEG1* is implicated in various morphological (tendon fiber diameter, bone porosity) and functional (strength) properties. Given that *TIEG1* is highly expressed in skeletal muscle [3], and in order to have a complete understanding of the effects of *TIEG1* on the musculo-skeletal system (i.e bone, tendon and muscle tissues), we sought to determine the biological roles of *TIEG1* in skeletal muscle.

A recent study has revealed that deletion of the *TIEG1* gene leads to changes in muscle structural properties [14]. Indeed, the MRI-TA (Magnetic Resonance Imaging—Texture Analysis) technique was applied to slow (soleus) and fast (EDL) twitch muscles isolated from the hind limb to characterize their texture properties. The results demonstrated different texture profiles for both muscles as a function of the mouse genotype (control *vs TIEG1*^-^/^-^). Moreover, these textural changes were accompanied by hypertrophy and hyperplasia in *TIEG1*^-^/^-^ mice. All of these structural modifications suggest that *TIEG1* plays an important role in skeletal muscle architecture and function.

Mechanical properties (stress, viscosity) of muscle fibers have been determined in different species such as rabbit [15], mice [16], rat [17], and cheetah [18]. Depending on the composition of muscle myofibrils (actin, myosin, titin, etc.), different tests (ramp stretch, relaxation, addition of calcium, etc…)[17] were applied to skinned or intact muscle fibers. Such samples demand high precision. Many studies have used a new generation of hardware (Aurora Scientific) to perform mechanical tests on single muscle fibers.

In addition to the experimental mechanical test, various mathematical models have been used to estimate the viscoelastic properties of the muscle fibers. In the literature, current approaches use linear models [19], quasi-linear viscoelastic models [19, 20] or hyperelastic models [21] to characterize the non-linear behavior of muscle fibers.

Given that *TIEG1* is most highly expressed in skeletal muscle [1], and based on our previous study demonstrating structural changes in the skeletal muscle of *TIEG1*^-^/^-^ mice [14], we hypothesized that TIEG1 plays a role in the contractile behavior of muscle fibers. Thus, the purpose of this study lies in its use of immunohistological analysis to better understand the classification of the different types of myosin heavy chain (MyHC) isoforms within the muscle and in the measurement of functional properties of skeletal muscle affected by *TIEG1* deletion. Additionally, we sought to analyze the effect of *TIEG1* deletion on the passive mechanical properties of slow (soleus) and fast (extensor digitorum longus: EDL) twitch muscle fibers. Here, we have employed various mechanical tests (ramp stretch, relaxation test) and a mathematical rheological model (3^rd^ order Hill) to assess the impact of *TIEG1* on the mechanical properties of skeletal muscle.

## Materials and Methods

### Muscle fiber preparation

Muscle fibers were extracted from a slow oxidative soleus muscle (Sol) and a fast glycolytic extensor digitorum longus (EDL) muscle [22, 23]. These two skeletal muscles were chosen based on their known differences in muscle fiber composition (Sol: types I, IIA, IIX and EDL: types IIB, IIX, IIA).

Soleus and EDL muscles were removed from the right hind limb of five *TIEG1*^−^/^−^ and five control (WT: Wild-Type) C57BL/6 female mice, sacrificed at 12 weeks of age by a CO_2_ exposure and cervical dislocation. Female mice were chosen for analysis in the present report due to previous publications demonstrating significant bone [24] and tendon [13] phenotypes in female animals. Experimental procedures and animal handling were done according to the French animal protection legislation including licensing of experimenters. They were reviewed and approved by the french veterinary services and the ethics commitee, named: Commission française de Génie Génétique (aggreement number #4635). All efforts made to minimize suffering. All muscles (*TIEG1*^−^/^−^: N_Sol_ = 5, N_EDL_ = 5; WT: N_Sol_ = 5, N_EDL_ = 5) were subjected to a specific preparation to extract fibers with a skinned membrane (sarcolema). Muscles were permeabilized at 4°C in a skinning solution (70 mM potassium propionate, 8 mM magnesium acetate, 5 mM EGTA, 7 mM ATP, 6 mM Imidazole, 10 mM PMSF, 50 mg.L^-1^ Trypsin inhibitor, 4 mg.L^-1^ Leupeptine, pH = 7.1). Different percentages of glycerol (12.5, 25 and 50%) were then added to the skinning solution [15, 18]. The permeabilized fiber bundles were stored in 50% glycerol skinning solution at -20°C.

### Passive mechanical tests

Under a binocular microscope (Leica M80), five single muscle fibers were isolated for each *TIEG1*^−^/^−^ (N_Sol_ = 25, N_EDL_ = 25) and WT (N_Sol_ = 25, N_EDL_ = 25) muscle. The fiber was then placed in a small bath (L = 14 mm) filled with a relaxing solution, corresponding to the skinning solution, at a control temperature of 25°C. An aluminum T-clip (Photofabrication, St Neots, UK) was used to connect the extremities of the fiber to a force transducer (5 mN) and to a motor (1400A - 802D Permeabilized Fiber Test Apparatus- Aurora Scientific). An imaging development system (IDS) camera (UI-1220LE, 752 x 480, 0.36 Mpx) allowed for visualization of the fiber architecture composed of successive sarcomeres. The distance between sarcomeres was measured using a Fourier transform algorithm [25].

Immediately prior to mechanical testing, each fiber was manually stretched to its initial length. This slack length (L_s_) was measured using an optical microscope (Leica DM IL LED) at a magnification of 20x. In addition, an average fiber diameter was determined using several measurements along the fiber length. Subsequently, each fiber underwent two preconditioning tests followed by two passive mechanical (ramp stretch, relaxation) tests.

**Fig 1:**
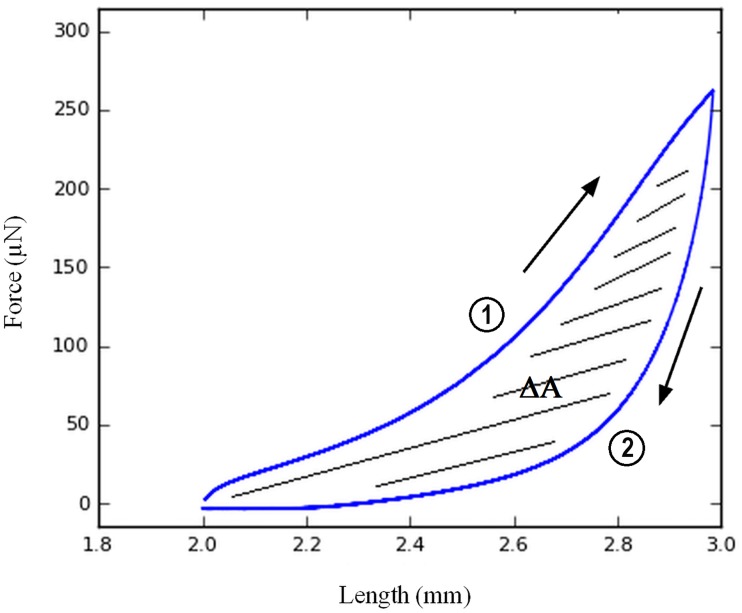
Representative force-length curve measurement. Hysteresis ΔA (hatched area) was calculated as the difference between the areas under the extension curve ① (arrow from left to right) and the de-stretch curve ② (arrow from right to left).

**Fig 2:**
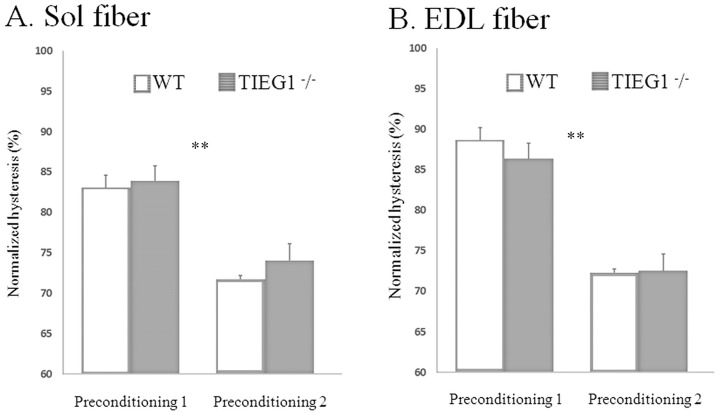
Normalized hysteresis ± SEM values measured after the two preconditioning tests performed on Sol and EDL fibers for each genotype. Soleus: Sol, extensor digitorum longus: EDL, WT: wild type, *TIEG1*^-^/^-^: Transforming Growth Factor β inducible early gene-1.

Preconditioning tests are often performed to insure realignment between all the myofibrils within the fiber, leading to a similar initial configuration across all muscle fibers before loading [16]. Accordingly, each fiber was stretched to 150% L_s_ at a lengthening rate of 0.005 L_s_/s and de-stretched at the same velocity. After a 5 minute rest period at the slack length, this preconditioning test was repeated. The hysteresis loop was plotted for each preconditioning run (Fig 1). The hysteresis area (ΔA) was calculated by subtracting the areas under the extension and de-stretch curves and the data was normalized to the area under the extension curve. The decrease of the hysteresis value (Fig 2) allows for controlling and validating the efficiency of the preconditioning.

The first passive mechanical test that was applied after preconditioning was a ramp stretch. The fibers were stretched to 150% L_s_ with a velocity of 0.1 L_s_/s allowing the measurement of the static force (Fs) at the end of the stretch. The second passive mechanical test performed was a relaxation test. Over a period of 60 s the fiber was repeatedly stretched to 150% L_s_ at high velocity (10 L_s_/s) and released to its slack length. The dynamic force (Fd), corresponding to the maximal force value, and the static force (Fs) measured at the end of the test were recorded.

**Fig 3:**
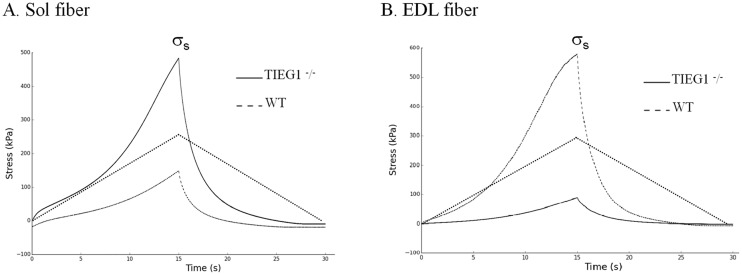
Experimental mechanical behavior of the Sol and EDL fibers, for each genotype, after a ramp stretch. The static stress (σs) was measured at the end of the stretch. Soleus: Sol, extensor digitorum longus: EDL, WT: wild type, *TIEG1*^-^/^-^: Transforming Growth Factor β inducible early gene-1.

All measured forces were divided by the cross-sectional area of the fiber to obtain dynamic (σ_d_) and static (σ_s_) stresses (Fig 3). Both mechanical tests were repeated two times for each fiber.

### Modeling the relaxation response

Based on the relaxation test that was performed at a single velocity (10 L_s_/s), a viscoelastic Hill model of 3^rd^ order [21] was used as a first step to fit the experimental viscoelastic data of muscle fiber response in a *TIEG1* knockout mouse model system. This model is composed of four branches: three branches include a dashpot of viscosity (c_i_) in series with a spring of stiffness (k_si_) while the fourth branch consists of a spring only that provides time-invariant material stiffness (k_p_). Stress (σ_M_) at time t can be expressed as a function of stretch amplitude (ε_r_) as follows:

$$\sigma_{M}\left(t\right)=\sigma_{s}+k_{s1}\varepsilon_{r}\;e^{-t/\tau_{s1}}+k_{s2}\varepsilon_{r}\;e^{-t/\tau_{s2}}+k_{s3}\varepsilon_{r}\;e^{-t/\tau_{s3}}$$ (1)

where $\tau_{si}=\frac{c_{i}}{k_{si}}$ are relaxation times.

During the experimental relaxation test, measurements of the static stress (σ_s_) (stress at equilibrium at t = ∞), and the dynamic stress (σ_d_) (stress at time t = 0), were allowed to have the following expressions:

$$\sigma_{s}=\sigma_{M}\left(\infty \right)=k_{p}\varepsilon_{r}$$ (2)

$$\sigma_{p}=\sigma_{M}\left(0\right)=\sigma_{s}+k_{s1}\varepsilon_{r}+k_{s2}\varepsilon_{r}+k_{s3}\varepsilon_{r}$$ (3)

Subsequently, a least-squares minimization was applied to fit the model given the experimental stress values (σ(t), σ_s_ and σ_d_). Based on the literature [26], three relaxation times were fixed to 0.01 s, 0.1 s and 10 s. The values of the elasticity parameters k_s1_ and k_s2_ were optimized in the range [0, (σd − σ_s_) / ε_r_], and the elasticity parameter k_s3_ was deduced from Eq (3), in order to minimize the cost function:

$$\chi^{2}=\sum_{n=1}^{N}{\left[\sigma \left(t_{n}\right)-\sigma_{M}\left(t_{n}\right)\right]}^{2}$$ (4)

where N is the number of time measurements and σ the experimental stress.

In order to compare experimental and mathematical fits, the root relative mean square is evaluated as:

$$e=\sqrt{\frac{\sum_{n=1}^{N}{\left[\sigma \left(t_{n}\right)-\sigma_{M}\left(t_{n}\right)\right]}^{2}}{\sum_{n=1}^{N}{\left[\sigma_{M}\left(t_{n}\right)\right]}^{2}}}$$ (5)

and the quality of fit r^2^ is defined by:

$$r^{2}=1-\frac{\sum_{n=1}^{N}{\left[\sigma \left(t_{n}\right)-\sigma_{M}\left(t_{n}\right)\right]}^{2}}{\sum_{n=1}^{N}{\left[\sigma \left(t_{n}\right)-mean\left(\sigma \left(t_{n}\right)\right)\right]}^{2}}$$ (6)

Values of r^2^ range from -∞ (worst model) to 1 (best model).

### Immunohistochemical analysis

Following the mechanical tests, each fiber was removed and immunohistochemical analysis [27] was performed to identify the fiber type as slow (type I) or fast (type IIB). Specific anti-MyHC antibodies BA-D5, SC-71, and BF-F3 [28] were purchased from DSHB (Developmental Studies Hybridoma Bank, Univ of Iowa, USA) to identify the fiber types I, IIA and IIB, respectively. The reactivity of these antibodies have previously been validated on mouse muscle tissues [22]. The fiber types were identified using a fluorescence microscope (Leica DMI 6000B).

Subsequently, three serial transverse sections (thickness: 10μm) were performed for each muscle using a cryostat (Cryo-star HM 560, Microm International GmbH, Germany) at -20°C. The samples were mounted on a glass slide and stained using an immunohistochemical process. To detect slow and fast MyHC isoforms, a combination of only two anti-myosin heavy chain (MyHC) antibodies were applied using a semi-automated method [27].

The cross-sections were blocked using 5% bovine serum albumin (BSA) diluted in phosphate-buffered saline (PBS) for 10 min to eliminate non-specific binding. They were then incubated with purified primary antibodies in a humidified dark chamber for one hour at 37°C, followed by several washes in PBS and incubation with the secondary antibodies for 30min in a dark room at 37°C [29]. Each section was co-labeled using anti-MyHC and anti-laminin antibodies. For soleus muscle, the MyHC isoforms I and IIA were revealed using BA-D5 and SC-71 primary antibodies, respectively, and an Alexa Fluor 488 goat anti-mouse IgG secondary antibody (A11001, Invitrogen, Carlsbad, CA, USA, dilution 1/200). For EDL muscle, the MyHC isoforms IIA and IIB were depicted using SC-71 and BF-F3, respectively. For the BF-F3 antibody, we used an Alexa Fluor 546 anti-mouse IgM secondary antibody (A11010, Invitrogen; dilution 1/1000). The cell outline was stained using a rabbit anti-laminin primary polyclonal antibody (L9393 Sigma-Aldrich, St. Louis, MO, USA) and a goat anti-rabbit IgG Cy3-labeled secondary antibody (111-165-008, Jackson Immunoresearch Labs, West Grove, PA, USA, dilution 1/200). The sections were extensively washed in PBS and mounted using Fluoromount (F4680, Sigma-Aldrich). Finally, non-specific reactivity was tested by performing the same immunocytochemistry procedure described above in the absence of primary antibodies (anti-MyHC and anti-laminin).

### Image Acquisition and Analysis

The histological sections were observed under a fluorescence microscope (Olympus BX 51) using a magnification of 20x and an appropriate band-pass filter (Alexa 488: excitation filter 460–495, emission filter 510–550, dichromatic mirror 505LP; Cy3: excitation filter 575–625, dichromatic mirror 565LP) as described by Meunier et al. [29]. High-resolution grayscale images were acquired with a camera (Olympus cooled digital camera DP-72) and the Cell-F software (Olympus Soft Imaging Solutions, Münster, Germany). Within each serial section, three regions of interest were chosen and two images corresponding to the laminin and MyHCs were digitized. The potential presence of hybrid fibers coexpressing two or more MyHC isoforms was also analyzed. Using a combination of two antibodies within each region of interest, for the Sol and EDL muscles, only two types of fibers could be identified (Sol _type I_: BA-D5, Sol _type IIA_: SC-71, EDL _type IIA_: SC-71, EDL _type IIB_: BF-F3). The third type (Sol_type IIX_, EDL_type IIX_) corresponds to unstained fibers.

Image processing and image analysis were performed with the FibTypFluo software program developed in Visual Basic under the Visilog 6.9 Professional Software (Noesis, Gif-sur-Yvette, France). This software automatically calculates the percentage and mean cross sectional area (CSA) of each fiber type.

### Statistical Analyses

The XLSTAT software package was used to perform all statistical analyses:

1. Normalized hysteresis values between the two preconditioning tests, for each genotype (*TIEG1*^−^/^−^, WT) and type of fiber (I, IIB), were compared using a paired t test;
2. Mechanical parameters (σd, σs) between *TIEG1*^−^/^−^ and WT, for each muscle (Sol, EDL), were compared using an unpaired t test;
3. Fiber area and percentage across different fiber types (I, IIA, IIX, IIB) was analyzed with an unpaired t test.

All data were expressed as mean ± standard error of the mean (SEM) with a level of significance set at P < 0.05.

## Results

### Measurements of the sarcomere length and diameter

Sarcomere lengths, measured at the slack length, within Sol and EDL fibers were not significantly different between *TIEG1*^-^/^-^ and their controls. For the soleus, the mean sarcomere lengths were 2.64 ± 0.08 μm and 2.52 ± 0.08 μm for *TIEG1*^-^/^-^ and WT, respectively. No significant difference in sarcomere lengths were observed for either genotype in the EDL muscle: 2.74 ± 0.10 μm for *TIEG1*^-^/^-^ and 2.92 ± 0.06 μm for WT.

Muscle fiber diameters for the Sol and EDL *TIEG1*^-^/^-^ fibers were significantly (P<0.01) greater (D_Sol-/-_ = 37.5 ± 1.2 μm, D_EDL-/-_ = 39.1 ± 1.3 μm) than matching WT fibers (D_Sol_WT_ = 33.8 ± 1.1 μm, D_EDL__WT = 31.9 ± 1.4 μm).

### Experimental mechanical properties

Fig 2 shows the normalized hysteresis values for the two preconditioning tests. Values from the second test dropped similarly for both genotypes (*TIEG1*^-^/^-^, WT) and muscle types (Sol, EDL). The decrease in hysteresis was similar for both fiber types, ~ 10% Sol and ~ 20% EDL, while no significant differences were found between genotypes. To test the value of preconditioning, the normalized hysteresis values of the ramp stretch test were compared to those of the second preconditioning test and found to be similar. This confirms that the muscle fibers were correctly preconditioned prior to starting the mechanical tests.

Fig 3 illustrates the mechanical response of the muscle fibers using ramp stretch. The soleus fibers displayed significantly greater (P < 0.001) static stress (σ_s_) for the *TIEG1*^-^/^-^ mice (σ_s_ = 333.44 kPa) compared to WT controls (σ_s_ = 212.40 kPa). The opposite held true for EDL fibers where significantly (P < 0.001) higher static stresses were measured for WT fibers (σ_s_ = 286.67 kPa) compared to *TIEG1*^-^/^-^ fibers (σ_s_ = 155.24 kPa).

**Fig 4:**
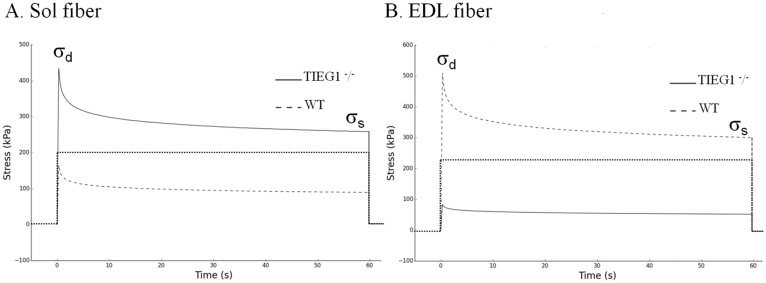
Experimental representations of the dynamic (σd) and static (σs) stresses obtained during the relaxation test performed on *TIEG1*^-^/^-^ and WT muscle fibers (Sol, EDL). Soleus: Sol, extensor digitorum longus: EDL, WT: wild type, *TIEG1*^-^/^-^: Transforming Growth Factor β inducible early gene-1.

On the relaxation test (Fig 4), the slow (soleus, type I) and the fast (EDL, type IIB) fibers revealed the same behavior as during the ramp stretch. The soleus *TIEG1*^-^/^-^ fibers exhibited significantly greater dynamic (P < 0.001) and static (P < 0.001) stresses (σ_d_ = 444.75 kPa, σ_s_ = 174.47 kPa) compared to the WT littermates (σ_d_ = 305.59 kPa, σ_s_ = 108.95 kPa). Inversely, the EDL *TIEG1*^-^/^-^ fibers displayed significantly (P < 0.001) decreased dynamic and static stresses (σ_d_ = 220.50 kPa, σ_s_ = 49.78 kPa) compared to the WT controls (σ_d_ = 438.62 kPa, σ_s_ = 102.79 kPa).

### Viscoelastic properties from the Hill model

**Fig 5:**
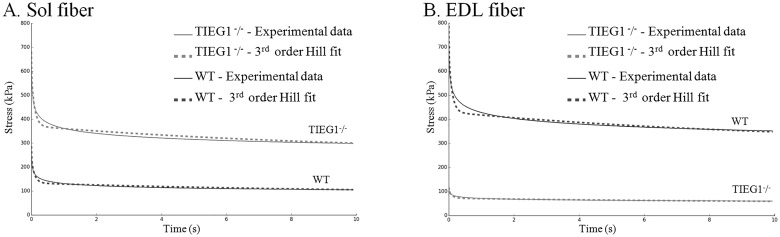
Comparison of experimental and numerical viscoelastic behaviors using a 3rd order Hill model for *TIEG1*^-^/^-^ and WT fibers (Sol, EDL). Soleus: Sol, extensor digitorum longus: EDL, WT: wild type, *TIEG1*^-^/^-^: Transforming Growth Factor β inducible early gene-1.

Fig 5 shows that a 3^rd^ order Hill model tracks well against the experimental curve for both genotypes (*TIEG1*^-^/^-^, WT) and fiber types (Sol, EDL), with low error percentage and high fit values (about 0.95) (Table 1).

**Table 1:** Numerical optimization results with SEM obtained with the 3^rd^ order Hill model fit.

Muscle	Mouse Type	Error value (%)	Fit value r^2^
**Sol**	*TIEG1*^-^/^-^	2.28 ± 0.09	0.949 ± 0.003
**Sol**	WT	2.59 ± 0.10	0.953 ± 0.003
**EDL**	*TIEG1*^-^/^-^	2.44 ± 0.10	0.948 ± 0.002
**EDL**	WT	2.64 ± 0.21	0.939 ± 0.006

Sol: soleus muscle. EDL: extensor digitorum longus muscle.

WT: wild type. *TIEG1*: Transforming Growth Factor β inducible early gene-1.

The different elastic parameters (k_p_, k_s1_, k_s2_, k_s3_) obtained for the Hill model are summarized in Table 2. The fiber relaxation behavior exhibited significant genotype dependence for the two types of fibers. Thus, the four elastic parameters of *TIEG1*^-^/^-^ Sol fibers were significantly (P < 0.02) greater than the WT parameters. Opposite results were obtained for the *TIEG1*^-^/^-^ EDL fibers which exhibited significantly (P < 0.003) decreased elastic properties compared to WT littermates.

**Table 2:** Elastic parameters with SEM calculated with the 3^rd^ order Hill model.

Muscle	Genotype	k_p_ (kPa)	k_s1_ (kPa)	k_s2_ (kPa)	k_s3_ (kPa)
**Sol**	*TIEG1*^-^/^-^	348.9 ± 31.4	165.0 ± 16.6	207.2 ± 19.7	168.3 ± 14.6
**Sol**	WT	217.8 ± 22.1	120.1 ± 10.6	155.1 ± 11.0	118.1 ± 10.2
**EDL**	*TIEG1*^-^/^-^	162.4 ± 11.5	80.4 ± 7.1	108.6 ± 8.6	86.5 ± 5.7
**EDL**	WT	295.5 ± 39.8	147.2 ± 18.3	195.3 ± 23.6	155.8 ± 17.6

Sol: *soleus* muscle. EDL: *extensor digitorum longus* muscle.

WT: wild type. *TIEG1*: Transforming Growth Factor β inducible early gene-1.

Among the four elasticities, the k_p_ parameter values (t = 0) were observed to be the most dominant (k_p_Sol_^-^_/_^-^ = 348.9 ± 31.4 kPa). It should also be noted that k_s2_ is the second most dominant parameter, indicating that a relaxation time of 100 ms best characterizes the relaxation behavior of the fibers compared to the other two relaxation times.

To model the relaxation response, the three relaxation times ($\tau_{si}=\frac{c_{i}}{k_{si}}$) were fixed by keeping the ratio of serial viscosity to serial elasticity constant. The viscosity of *TIEG1*^-^/^-^ Sol fibers was significantly greater (c_2_ = 20.7 ± 1.9 kPa.s; P < 0.003) than the WT Sol fibers (c_2_ = 15.5 ± 1.1 kPa.s). For the *TIEG1*^-^/^-^ EDL fibers the reverse phenomenon was observed with decreased (c_2_ = 10.9 ± 0.8 kPa.s) viscosity compared to WT animals (P < 0.003).

### Immunohistochemical staining

**Fig 6:**
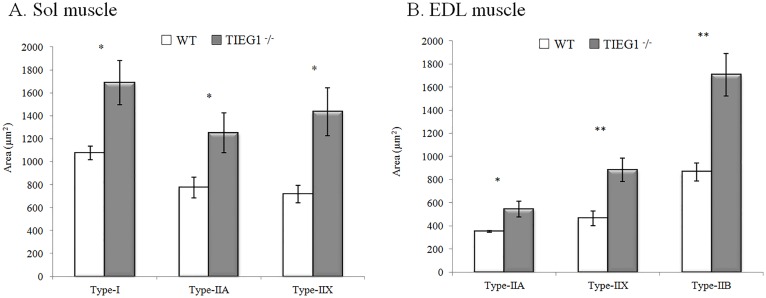
(A) Sol and (B) EDL fiber areas (mean ± SEM) for *TIEG1*^-^/^-^ and WT mice. Soleus: Sol, extensor digitorum longus: EDL, WT: wild type, *TIEG1*^-^/^-^: Transforming Growth Factor β inducible early gene-1. Signification:*: P<0.05; **: P<0.01.

**Fig 7:**
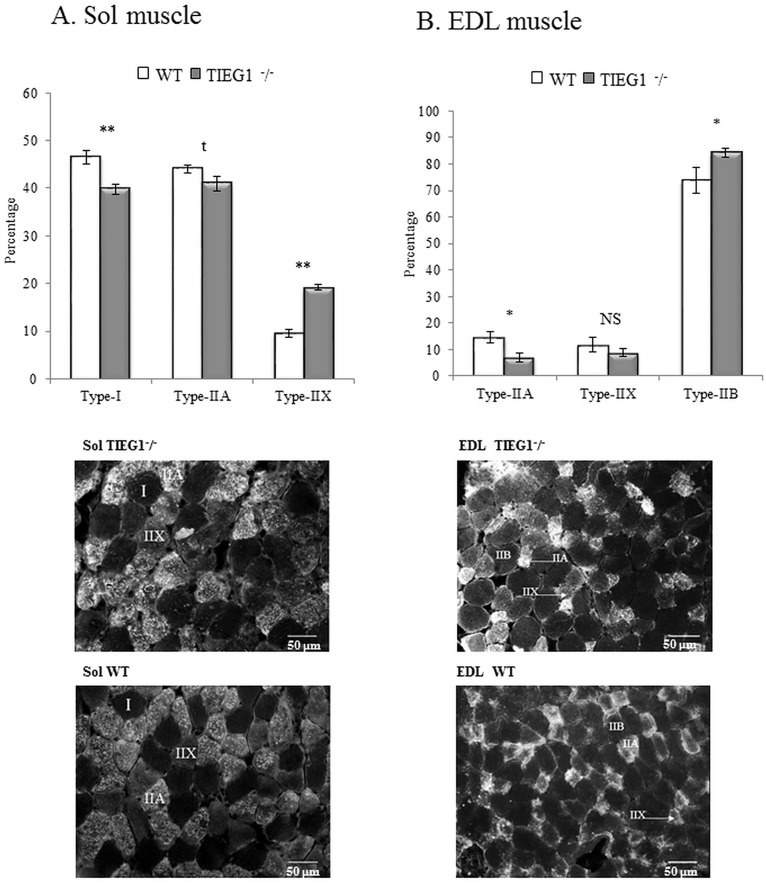
Percentage of fiber type (I, IIA, IIB, IIX) within Sol (A) and EDL (B) muscles for both genotypes (*TIEG1*^-^/^-^,WT). Representation of the fiber types through immunohistological staining. Soleus: Sol, extensor digitorum longus: EDL, WT: wild type, *TIEG1*^-^/^-^: Transforming Growth Factor β inducible early gene-1. Signification: *: P<0.05; **: P<0.01; NS: Not significant; t: Tendency.

The results obtained from immunohistochemical staining did not indicate the presence of hybrid fibers in either the soleus or EDL muscles for wild-type or *TIEG1*^-^/^-^ mice. A significant increase (P < 0.05) in all *TIEG1*^-^/^-^ fiber areas (I, IIA, IIX, and IIB) compared to the WT fiber areas (Fig 6A and 6B). As shown in Fig 7A and 7B, the percentage of the different fiber types present within the Sol (I, IIA, and IIX) and EDL (IIB, IIA, and IIX) muscles are indicated. *TIEG1*^-^/^-^ Sol muscle (Fig 7A) revealed a significant (P < 0.01) decrease of type I and a significant (P < 0.01) increase of type IIX fibers compared to WT Sol muscle. *TIEG1*^-^/^-^ EDL muscles (Fig 7B) showed a significantly (P < 0.05) lower percentage of type IIA and a significantly (P < 0.05) higher percentage of type IIB compared to WT EDL muscle. No significant differences were observed across genotypes in the percentage of type IIA fibers within Sol muscles or in the percentage of type IIX within EDL muscles.

## Discussion

This study follows our recent work to characterize the morphological properties of *TIEG1*^-^/^-^ skeletal muscles [14]. We have demonstrated that the deletion of *TIEG1* results in the hyperplasia and hypertrophy of slow (soleus) and fast (EDL) muscles. In the present study, using immunohistological analyses, we have complemented the previous results by revealing hypertrophy of all fiber types (I, IIA, IIX, and IIB) present within the Sol and EDL muscles of *TIEG1*^-^/^-^ mice. No hybrid fiber was identified in both muscles and it is well known that they are occasionally (less than 2%) present in adult mice [22, 27, 30]. Even though, hybrid fibers were not depicted the hypertrophy was revealed for all types of myosin. A similar hypertrophy of tissues has been observed previously in *TIEG1*^-^/^-^ tendon fibers [13] and *TIEG1*^-^/^-^ heart tissues [31]. Future studies, aimed at specifically deleting the *TIEG1* gene in muscle tissue will allow us to eliminate potential confounding effects relating to the known defects in *TIEG1*^-^/^-^ mouse bone and tendon tissue.

In addition to the hypertrophy phenotype, an analysis of the percentage of fiber types revealed that the slow (Sol) and fast (EDL) *TIEG1*^-^/^-^ muscles have more glycolytic (IIX and IIB) fibers and less oxidative (I and IIA) fibers compared to WT muscles. It can be concluded that the deletion of *TIEG1*^-^/^-^ led to glycolytic hypertrophy, regardless of the muscle type. A previous publication has demonstrated that *TIEG1* expression increases during the differentiation of C2C12 cells into myoblasts [32]. Additionally, Miyake et al. have demonstrated that suppression of *TIEG1* expression in C2C12 cells results in increased cell proliferation, myotube formation and fusion. This result is in agreement with the hyperplasia and hypertrophy found in *TIEG1*^-^/^-^ muscles in the present study. Taken together, these results suggest that *TIEG1* negatively impacts muscle cell differentiation. Furthermore, it is well known that myostatin (a member of the TGF-β superfamily) is implicated in the regulation of muscle hypertrophy [33]. Overexpression of myostatin and TGF-β are known to induce *TIEG1* expression in C2C12 cells [34]. It is therefore possible that *TIEG1* is a downstream mediator of myostatin function in muscle cells and that the muscle fiber hypertrophy observed in the present study is due to defects in myostatin signaling. It will be of interest further analyze the basis for glycolytic hypertrophy through the use of gene and protein expression profiling and to explore potential alterations in titin expression which may play a role in hypertrophic signaling [35].

Two mechanical tests (ramp stretch and relaxation test) were chosen as a first step to characterize the role of *TIEG1* on the mechanical properties of slow and fast twitch muscle fibers. Although a preconditioning test was applied to avoid the effects of past loading, the slow (Sol) and fast (EDL) fiber genotypes produced similar normalized hysteresis data. This result implicates that deletion of *TIEG1* has no effect on the preconditioning of the muscle fibers regardless of the fiber type (slow *vs* fast). Deletion of the *TIEG1* gene does, however, lead to significant changes in mechanical properties: an increase in the static and dynamic stresses for *TIEG1*^-^/^-^ Sol fibers (type I) and a decrease for the *TIEG1*^-^/^-^ EDL fibers (type IIB) compared to WT littermates. These modifications are not related to sarcomere length given that similar ranges were measured between *TIEG1*^-^/^-^ and WT fibers. It will of interest to examine changes in titin expression in *TIEG1*^-^/^-^ mice which is known to be involved in the myofilament distensibility in skeletal muscle [35]. In the literature, functional modifications have been identified for *TIEG1*^-^/^-^ tendon fibers [13] and *TIEG1*^-^/^-^ cortical bone [12]. From the mechanical properties, it can be concluded that the effect of *TIEG1* is fiber type-specific (slow *vs* fast) which may be explained by differences in the metabolic (glycogen, oxygen) and contractile properties (type of myosin, actin, titin) that are known to exist between these two types of fibers [22, 23]. This may be achieved through compensatory changes within the muscles to maintain the homeostasis of the whole muscle during postnatal life. The present mechanical analysis should not be limited to passive test (stretch) but also performed under active conditions by inducing a contraction within the muscle.

The modeling response of the experimental relaxation curve with the use of a 3^rd^ order Hill model has improved our understanding of the viscous behavior through measurements of k_p_ and k_s2_. The main limitation of the present method is that it does not incorporate nonlinear viscosities. Indeed, our relaxation experiment was performed at a single velocity because the main objective was to investigate the mechanical properties between *TIEG1*^-^/^-^ and WT fibers extracted from slow and fast twitch muscles. In perspective, we could have analyzed the dependency on the stretching velocity to capture the effects of nonlinear viscosities. For that purpose, other approaches such as those based on a quasi-linear model [19, 20], or a hyperelastic model [21] would need to be employed. The Hill model presented here, though, has the advantage of requiring only two parameters to fit the experimental curve while more parameters are needed in the other models.

It can be concluded that deletion of *TIEG1*^-^/^-^ leads to glycolytic muscle hypertrophy accompanied by an increase in mechanical stresses in type I Sol fibers and a decrease in stresses in type IIB EDL fibers. Further investigation of the muscle metabolism is necessary to better understand the molecular basis for the observed defects in the skeletal muscles of *TIEG1*^-^/^-^ mice and will allow us to better translate these finding to human myopathies.
